# Differential Roles of Actin Crosslinking Proteins Filamin and α-Actinin in Shear Flow-Induced Migration of *Dictyostelium discoideum*

**DOI:** 10.3389/fcell.2021.743011

**Published:** 2021-08-16

**Authors:** Aaron Cole, Sarah Buckler, Jack Marcucci, Yulia Artemenko

**Affiliations:** Department of Biological Sciences, State University of New York Oswego, Oswego, NY, United States

**Keywords:** directed migration, mechanotransduction, signal transduction network, mechanical perturbation, shear stress, cell cortex

## Abstract

Shear flow-induced migration is an important physiological phenomenon experienced by multiple cell types, including leukocytes and cancer cells. However, molecular mechanisms by which cells sense and directionally migrate in response to mechanical perturbation are not well understood. *Dictyostelium discoideum* social amoeba, a well-established model for studying amoeboid-type migration, also exhibits directional motility when exposed to shear flow, and this behavior is preceded by rapid and transient activation of the same signal transduction network that is activated by chemoattractants. The initial response, which can also be observed following brief 2 s stimulation with shear flow, requires an intact actin cytoskeleton; however, what aspect of the cytoskeletal network is responsible for sensing and/or transmitting the signal is unclear. We investigated the role of actin crosslinkers filamin and α-actinin by analyzing initial shear flow-stimulated responses in cells with or without these proteins. Both filamin and α-actinin showed rapid and transient relocalization from the cytosol to the cortex following shear flow stimulation. Using spatiotemporal analysis of Ras GTPase activation as a readout of signal transduction network activity, we demonstrated that lack of α-actinin did not reduce, and, in fact, slightly improved the response to acute mechanical stimulation compared to cells expressing α-actinin. In contrast, shear flow-induced Ras activation was significantly more robust in filamin-null cells rescued with filamin compared to cells expressing empty vector. Reduced responsiveness appeared to be specific to mechanical stimuli and was not due to a change in the basal activity since response to global stimulation with a chemoattractant and random migration was comparable between cells with or without filamin. Finally, while filamin-null cells rescued with filamin efficiently migrated upstream when presented with continuous flow, cells lacking filamin were defective in directional migration. Overall, our study suggests that filamin, but not α-actinin, is involved in sensing and/or transmitting mechanical stimuli that drive directed migration; however, other components of the actin cytoskeleton likely also contribute to the initial response since filamin-null cells were still able to activate the signal transduction network. These findings could have implications for our fundamental understanding of shear flow-induced migration of leukocytes, cancer cells and other amoeboid-type cells.

## Introduction

Directed migration of cells to cues from their environment plays an important role in diverse physiological and pathophysiological processes, including embryo development, inflammation and cancer metastasis (Barriga and Theveneau, 2020; SenGupta et al., 2021). The best understood mode of directed cell migration is chemotaxis, where cells detect a chemical gradient in the environment and migrate relative to that gradient. However, other environmental conditions, such as substrate stiffness (durotaxis), electric fields (galvanotaxis), and physical forces, such as shear flow, can also guide migration, although the molecular mechanisms of these types of directed migration are not well understood (Shellard and Mayor, 2020).

*In vivo* cells experience a variety of shear forces depending on the anatomical location. In addition to the shear forces experienced by cells within blood and lymphatic vessels, ranging from < 1 to 12 dyn/cm^2^ in the lymphatic system, up to 6 dyn/cm^2^ in the veins, and 10–70 dyn/cm^2^ in the arteries, most cells are also subject to interstitial fluid flow caused by plasma that leaks out of the capillaries and drains through the tissue to the lymphatic system (Malek et al., 1999; Wiig and Swartz, 2012). Although shear stress values generated by interstitial fluid flow are small under physiological conditions (<0.1 dyn/cm^2^), lymphatic and interstitial flow is increased in the stroma of most tumors because of the leaky vasculature, and this can influence multiple processes in the tumor microenvironment, including adhesion and migration of fibroblasts and tumor cells (Swartz and Lund, 2012). Recent studies have shown that shear flow promotes migration of various cancer cell lines and can even induce changes from mesenchymal to amoeboid-type migration of breast cancer cells in a 3D matrix (Qazi et al., 2013; Huang et al., 2015). Shear flow is also critical for migration of leukocytes following arrest within the blood vessel, as well as for transmigration (Valignat et al., 2013; Fine et al., 2016). Somewhat surprisingly, the direction of cell migration in response to shear flow appears to be dependent on a variety of factors and migration can occur either upstream or downstream of the flow depending on the type of cells, the types of adhesion molecules they express, and the strength of the shear stress (Fache et al., 2005; Valignat et al., 2013; Dominguez et al., 2015; Artemenko et al., 2016; Buffone et al., 2018, 2019).

Social amoeba *Dictyostelium discoideum* has been instrumental in understanding the biochemical and molecular pathways that control directional cell movement (Devreotes et al., 2017; Stuelten et al., 2018; Bozzaro, 2019). The amoeboid-type migration of single *D. discoideum* cells, characterized by fast speed and weak interaction with the substrate, is remarkably similar to the migration of mammalian cells, including neutrophils, stem cells and metastatic cancer cells (Paluch et al., 2016). Importantly, both the overall organization of the regulatory networks, as well as individual signal transduction pathways, are conserved between *D. discoideum* and mammalian cells, such as leukocytes (Artemenko et al., 2014). *D. discoideum* relies on directed migration to chemoattractants throughout its unique life cycle (Bozzaro, 2019). During the single-cell growth (vegetative) stage, *D. discoideum* sense chemoattractants to locate food sources. When food becomes scarce, cells initiate a developmental program that allows thousands of cells to chemotax toward each other, eventually forming multicellular aggregates that differentiate to produce a fruiting body with spores. However, given the diverse environment of the forest floor, where *D. discoideum* is naturally found, it is not surprising that these cells are also able to respond and directionally migrate to other stimuli, including shear flow, making this organism an excellent model system for investigating the molecular mechanisms of this process.

Unlike chemotaxis, where the process is initiated by binding of a chemoattractant to a receptor, how mechanical stimuli are detected and converted to a biochemical response that leads to directed migration is less clear. Mechanical perturbation could cause physical changes in the interaction between the cell and the substrate, induce ion channel opening, affect the linkage between the membrane and the cytoskeleton, and/or perturb the cytoskeleton itself. In particular, integrins and mechanosensitive ion channels have been implicated as important mechanotransducers in a variety of cell types, although relatively few studies examined the mechanism of mechanotransduction in shear flow-induced migration (Ranade et al., 2015; Han and de Rooij, 2016). In *Dictyostelium* and some mammalian cells, Ca^2+^ appears to be an important regulator of shear flow-induced motility (Fache et al., 2005; Zhu et al., 2014; Gao et al., 2019). Consistent with the role of calcium in this type of directed migration, Lima et al. (2014) demonstrated that PKD2, a homolog of human polycystin-2 component of the mechanosensitive cation channel PKD, is required for migration of vegetative *Dictyostelium* cells in response to shear flow, although another mechanosensitive channel MclN might also play a minor role (Lima et al., 2014). Interestingly, studies in T lymphocytes, whose direction of migration depends on whether they express integrins that can bind to ICAM or LFA-1, might not have specific mechanosensors and their shear flow-induced migration appears to be determined by passive steering by the non-adherent part of the cell (Valignat et al., 2013, 2014; Hornung et al., 2020).

Although the initial steps in sensing mechanical cues are not clear, previous research has shown that shear flow activates the same signal transduction network that is activated by chemoattractants (Artemenko et al., 2016). This excitable signal transduction network consists of multiple parallel and interconnected pathways that ultimately feed into the actin cytoskeletal network, biasing actin polymerization and pseudopod protrusion in the direction of the guidance cue (Devreotes et al., 2017). The polarized distribution of signal transduction network components defines the leading and lagging edges of the cell, with similar distribution seen at the tip and bases of individual protrusions. Importantly, uniform stimulation with a chemoattractant or brief stimulation with shear flow leads to a transient global response of the signal transduction and cytoskeletal networks, which can be observed by accumulation and/or activation of leading edge markers, such as Ras, phosphatidylinositol 3-kinase (PI3K) and actin polymerization, at the cell cortex. Concomitantly, back markers, such as PTEN and myosin II, relocalize from the cortex to the cytosol. When cells are first introduced to a gradient of a chemoattractant or to shear flow, biased signal transduction and cytoskeletal network activity is observed only after the initial global response (Artemenko et al., 2016). Thus, measurement of the initial response allows us to gain insight into the ability of the cell to sense and respond to the stimulus.

One clear distinction between chemical and mechanical stimulus sensing is that cells treated with latrunculin A, which disrupts the actin cytoskeleton, are unable to activate their signal transduction network in response to shear flow, but can do so in response to a chemoattractant (Parent et al., 1998; Artemenko et al., 2016; Pal et al., 2019). This suggests that an intact actin cytoskeleton is required for sensing or transmitting mechanical stimuli; however, what aspect of the cytoskeleton is involved in this process is unclear. The importance of the actin cytoskeleton in this system suggests that actin-associated proteins may bridge the gap between mechanical stimulation and a biochemical response.

Several actin crosslinkers, including filamin and α-actinin, have been implicated in mechanosensation downstream of integrins in mammalian cells, as well as in cortical mechanosensing independent of adhesion (Han and de Rooij, 2016; Schiffhauer and Robinson, 2017). Both filamin and α-actinin possess an actin-binding domain (ABD) as well as a dimerization domain responsible for mediating formation of Y-shaped or anti-parallel dimers, respectively. While α-actinin bundles actin filaments tightly and crosslinks them in a parallel orientation, filamin crosslinks actin filaments together orthogonally (Razinia et al., 2012; Murphy and Young, 2015). Mechanical stress applied to filamin or α-actinin reveals cryptic binding sites that provide alternate binding locations, for example for integrin or vinculin, while under mechanical stress (Razinia et al., 2012; Le et al., 2017). Importantly, filamin and α-actinin accumulate at different zones of stress in response to mechanical stimuli delivered by micropipette aspiration (Luo et al., 2013), suggesting they likely have divergent functions in sensing or transmitting mechanical stimuli.

Unlike multiple isoforms of filamin and α-actinin found in mammalian cells, *D. discoideum* has a single isoform of each crosslinker. Mutant strains lacking *D. discoideum* filamin (ddFLN, ABP120, gelation factor) or α-actinin (ddACTN) did not have strong phenotypes on overall behavior at the single cell stage, including growth, development, random motility and chemotaxis, although more severe defects were observed when ddFLN was deleted in a different genetic background (Schleicher et al., 1988; Brink et al., 1990; Cox et al., 1992; Rivero et al., 1996). This could be due to functional redundancy of the crosslinkers, since mutants lacking both ddFLN and ddACTN had severe defects in growth, development, and migration (Witke et al., 1992; Rivero et al., 1996). Despite the minor differences in behavior in the original null strains, absence of individual crosslinkers led to changes in the viscoelastic properties of the cells (Eichinger et al., 1996). Increased levels of actin crosslinkers likely also have effects on cortical stiffness and could explain why overexpression of ddFLN adversely affects cell migration, particularly in a 3D environment (Roth et al., 2015).

Although both filamin and α-actinin have been implicated in mechanosensation, their role in regulating shear flow-induced migration has remained largely unexplored. Thus, in this study we investigated the role of filamin and α-actinin in the initial response of *D. discoideum* cells to brief stimulation with shear flow and demonstrated that only filamin appears to participate in sensing and/or transmitting the mechanical stimulus in this system. Consistent with the requirement of the initial response for shear-flow induced migration under continuous flow, cells lacking filamin had aberrant directed migration against the flow direction compared to cells with filamin, further implicating this actin crosslinker as an important player in this mechanically-driven form of directed migration.

## Materials and Methods

### Cell Culture and Generation of *D. discoideum* Strains

*D. discoideum* cells were maintained under standard conditions on plates or in shaking culture in HL-5 media (Artemenko et al., 2011). Wild-type (Ax2) cells were generously provided by R. Kay (MRC Laboratory of Molecular Biology, Cambridge, United Kingdom). α-actinin-null (*abpA*^–^; strain ID DBS0235459) and filamin-null (*abpC*^–^; strain ID DBS0236077) cell lines used in this study were obtained from the Dicty Stock Center (Chicago, IL, United States). Note that for clarity, *abpA*^–^ and *abpC*^–^ will be referred to as *actn*^–^ and *fln*^–^, respectively.

Ax2, *actn*^–^, or *fln*^–^ cells were transformed with RBD-GFP and/or mCherry-ddFLN, mCherry-ddFLN_ΔABD_, mCherry-ddACTN or pDRH by electroporation according to the standard electroporation protocol (Gaudet et al., 2007). Transformed cells were selected with 20 μg/ml G418 and/or 50 μg/ml hygromycin B for at least 2 weeks prior to analysis.

pDRH, mCherry-ddFLN in pDRH, and ddACTN-GFP in pDN plasmids were generously provided by D. Robinson (Johns Hopkins University). RBD-GFP construct in pDM323 was generously provided by P. Devreotes (Johns Hopkins University). mCherry-ddFLN_ΔABD_ and mCherry-ddACTN were generated in this study as described below.

### Plasmid Construction

To generate mCherry-ddACTN in the pDRH vector, the ddACTN gene was amplified from a pDN-ddACTN-GFP template by PCR utilizing Phusion High-Fidelity PCR Master Mix (Thermo Scientific). The forward and reverse primers used were 5′-GGCGTCGACTCAGAAGAACCAACCCCAG-3′ and 5′-ACTACAATTCATTTGCTGGCGGCCGCCC-3′, which incorporated *Sal*I and *Not*I restriction sites, respectively. pDRH-mCherry-ddFLN plasmid was digested with *Sal*I and *Not*I restriction enzymes (Thermo Scientific) to remove the ddFLN gene without the mCherry tag, dephosphorylated with FastAP thermosensitive alkaline phosphatase (Thermo Scientific), and purified with the GeneJET^TM^ gel extraction kit (Thermo Scientific). Following digestion with *Sal*I and *Not*I, ddACTN gene was ligated into the digested pDRH-mCherry fragment using T4 DNA ligase (Thermo Scientific). Ligation products were transformed into NEB^®^ 5-alpha competent *Escherichia coli* (high efficiency) cells (New England Biolabs) and selected on LB agar plates with 150 μg/mL ampicillin. Plasmids were isolated using the GeneJET^TM^ Plasmid Miniprep Kit (Thermo Scientific) and digested with *Sal*I and *Not*I to confirm successful ligation. Successful clones were verified by sequencing (Genewiz).

mCherry-ddFLN_ΔABD_ was generated by amplifying ddFLN starting at position 745 (corresponding to amino acid 249) based on the study by Washington and Knecht using 5′-GGCGTCGACGATGCCAGCAAGGTTGAAGTTTATGG-3′ and 5′-GGCGCGGCCGCTTAATTGGCAGTACGAGTAGTAG TG-3′ primers, which incorporated *Sal*I and *Not*I sites, respectively (Washington and Knecht, 2008). Following amplification, the insert was ligated into pDRH-mCherry as described above for the ddACTN construct.

### Preparation of Vegetative *D. discoideum* Cells

*D. discoideum* cells were grown in the presence of *Klebsiella aerogenes* on SM plates as described in Artemenko and Devreotes (2017). Briefly, *K. aerogenes* was grown in an antibiotic-free HL-5 medium overnight (16–18 h). 6 × 10^5^–1.2 × 10^6^ *D. discoideum* cells were added onto SM plates with 260 μL of the *K. aerogenes* suspension. The plates were incubated at room temperature for 2 days. To collect the cells, the lawn containing *K. aerogenes* and *D. discoideum* was scraped with a sterile spreader into DB buffer (1x phosphate buffer supplemented with 2 mM MgSO4 and 0.2 mM CaCl2), collected in a 50 mL polypropylene centrifuge tube, and centrifuged at 500 *g* for 3–4 min. Following several washes with DB, the pellet was resuspended to ∼5 × 10^6^ cells/mL.

### Mechanical Stimulation of *D. discoideum*

Vegetative cells collected from *K. aerogenes* lawn were diluted to ∼1 × 10^6^ cells/mL in DB, plated on a μ-Slide III^3in1^ fluidic chamber (Ibidi) and allowed to attach for 10 min. The fluidic slide was attached to the Ibidi pump system and the cells were briefly washed with DB buffer as previously described (Artemenko and Devreotes, 2017). Cells were imaged with epifluorescence with a GFP or an RFP filter on a Zeiss LSM 700 microscope equipped with a 63 × /1.3 oil objective lens and a Zeiss MRc AxioCam camera. Images were taken at 3 s intervals for 60 s. Immediately after the fifth frame, the cells were mechanically stimulated using the Ibidi pump at the indicated pressure for 2 s. The highest pressure applied was 50 mbar, which corresponded to ∼45 dyn/cm^2^ shear stress. We also used flow driven by gravity alone and varied the pressure by changing the height of the pump, which allowed us to generate shear stress values of ∼23, ∼17, ∼12 and ∼6 dyn/cm^2^. Shear stress values were calculated by measuring the volume of fluid collected in the drain over time and converting it to shear stress values using the formula provided by the manufacturer (Ibidi) for the 3 mm channel in the μ-Slide III^3in1^ fluidic chamber: τ = η × 227.4 × Φ, where τ is shear stress, η is the dynamic viscosity of the medium, which is the viscosity of water at 20°C (0.01 dyn⋅s/cm^2^), and Φ is the flow rate in mL/min. It should be noted that shear stress values are approximated, rather than exact, because there was slight variation (maximum of ∼4 dyn/cm^2^) between shear stress values depending on the volume of buffer in the reservoir; however, for the assays the fluctuation in the volume of buffer in the reservoir was kept to a minimum. In all of the images produced, flow was applied from right to left.

### Chemical Stimulation of *D. discoideum*

Vegetative cells were grown and collected from *K. aerogenes* lawn as described above. 1 × 10^5^ cells were plated in 450 μl of DB in 8-well Nunc^TM^ Lab–Tek^TM^ II chambered coverglass. Cells were imaged every 3 s for 20 frames with epifluorescence with a GFP or RFP filter set on a Zeiss LSM 700 microscope equipped with a 63 × /1.3 oil objective lens and a Zeiss MRc AxioCam camera. After frame 5, 50 μl of 1 mM folic acid was introduced to the cells.

### Image Analysis for Mechanical and Chemical Stimulation

Images were analyzed using Fiji (ImageJ 1.53c) software (Schindelin et al., 2012; Artemenko and Devreotes, 2017). Every cell image that was in focus was quantified, except for analysis of RBD-GFP signal in rescue cells, where only cells expressing mCherry-ddFLN, mCherry-ddFLN_ΔABD_ or mCherry-ddACTN were quantified. The background was subtracted, and a box was drawn within the cytoplasm of a given cell. The mean intensity inside this box was quantified for every frame produced. Care was taken to avoid areas within the cell with a visible nucleus or a concentration of vesicles as this would provide false drops in levels of cytoplasmic intensity. The quantified levels of cytosolic intensity were normalized for time 0 and inverted to provide values of cortical accumulation. Note that due to slight transient shift in the focal plane that was occasionally observed for the frame immediately following the stimulation, the 3 s time point was omitted from the analysis.

### Migration Assay

To measure cell movement, both randomly and in response to shear flow, vegetative cells grown with *K. aerogenes* were collected, washed, and plated as described above for mechanical stimulation experiments. Cells were imaged with brightfield illumination using a Zeiss LSM 700 confocal microscope equipped with a 20X objective lens and a Zeiss MRc AxioCam camera. Time lapse images were taken every 10 s. To quantify random migration, cells were imaged for 60 frames in the absence of flow. To measure directed migration, cells were subjected to continuous flow for 120 frames using the Ibidi pump with an open reservoir. For this assay 6, 12, or 17 dyn/cm^2^ shear stress value was used.

50 randomly selected cells were tracked for each experiment with the Tracking Tool^TM^ PRO v2.1 software (Gradientech). For random migration, analysis was started 2 min after the beginning of the assay and proceeded for 5 min. For directed migration, analysis was started 2 min after shear flow was introduced and proceeded for 18 min. Velocity, directness, and forward migration index were calculated using TrackingTool Pro, which reports average values for the 50 cells analyzed. Velocity measures the overall cell displacement over time in μm/min. Directness is calculated as a ratio of the shortest distance between the start and end points of the path traveled to the total accumulated distance traveled by a cell. Forward migration index refers to the directness in the direction of the applied stimulus and is calculated using the end point in the *x* direction divided by accumulated distance. In this case flow was applied from right to left, so a forward migration index value of 1 would indicate migration directly against the flow and −1 would indicate migration with the flow.

### Statistical Analysis

An unpaired two-tailed, two-sample equal variance student’s *t*-test was used to analyze differences between cell lines at every time point of mechanical or chemical stimulation assays. A paired student’s *t*-test was used to test for differences between two cell lines for migration analysis. *P* < 0.05 was considered statistically significant.

## Results

### Filamin and α-Actinin Transiently Accumulate at the Cortex in Response to Mechanical Stimulation

**FIGURE 1:**
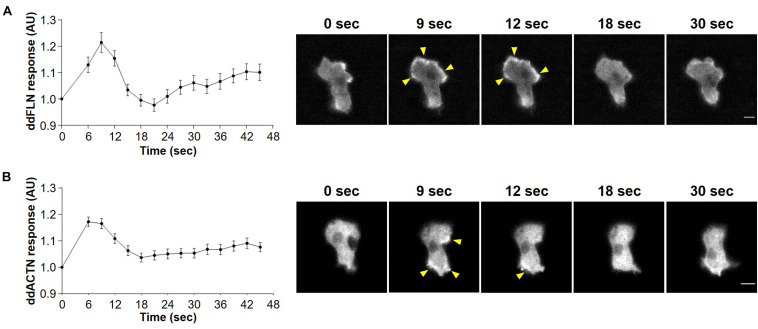
Localization of filamin and α-actinin in response to acute treatment with shear flow. Wild-type cells expressing mCherry-tagged ddFLN **(A)** or mCherry-tagged ddACTN **(B)** were imaged every 3 s by epifluorescence microscopy with an RFP filter set under 630X magnification with oil immersion. Shear flow at 45 dyn/cm^2^ was applied at time 0 for 2 s. Response was measured as protein relocalization from the cytosol to the cell cortex. Data is displayed as mean ± SE of 45 **(A)** or 85 **(B)** cells pooled from three independent experiments. A representative cell is shown to the right of the graph. Arrowheads point to mCherry-tagged protein accumulation at the cortex. Scale bar, 5 μm.

To determine whether actin crosslinking proteins ddFLN and ddACTN are involved in regulating the response of cells to shear flow, we first examined the behavior of these proteins following stimulation with shear flow. Both mCherry-tagged ddFLN and ddACTN transiently localized to the cell cortex of wild-type cells following 2 s stimulation with shear flow at 45 dyn/cm^2^ (Figure 1 and Supplementary Video 1). Cortical accumulation peaked at 6–9 s after shear flow stimulation, consistent with the timing of the peak response of other leading edge markers and actin polymerization at the cortex (Artemenko et al., 2016).

### Filamin Is Required for Optimal Response to Acute Mechanical Stimulation

**FIGURE 2:**
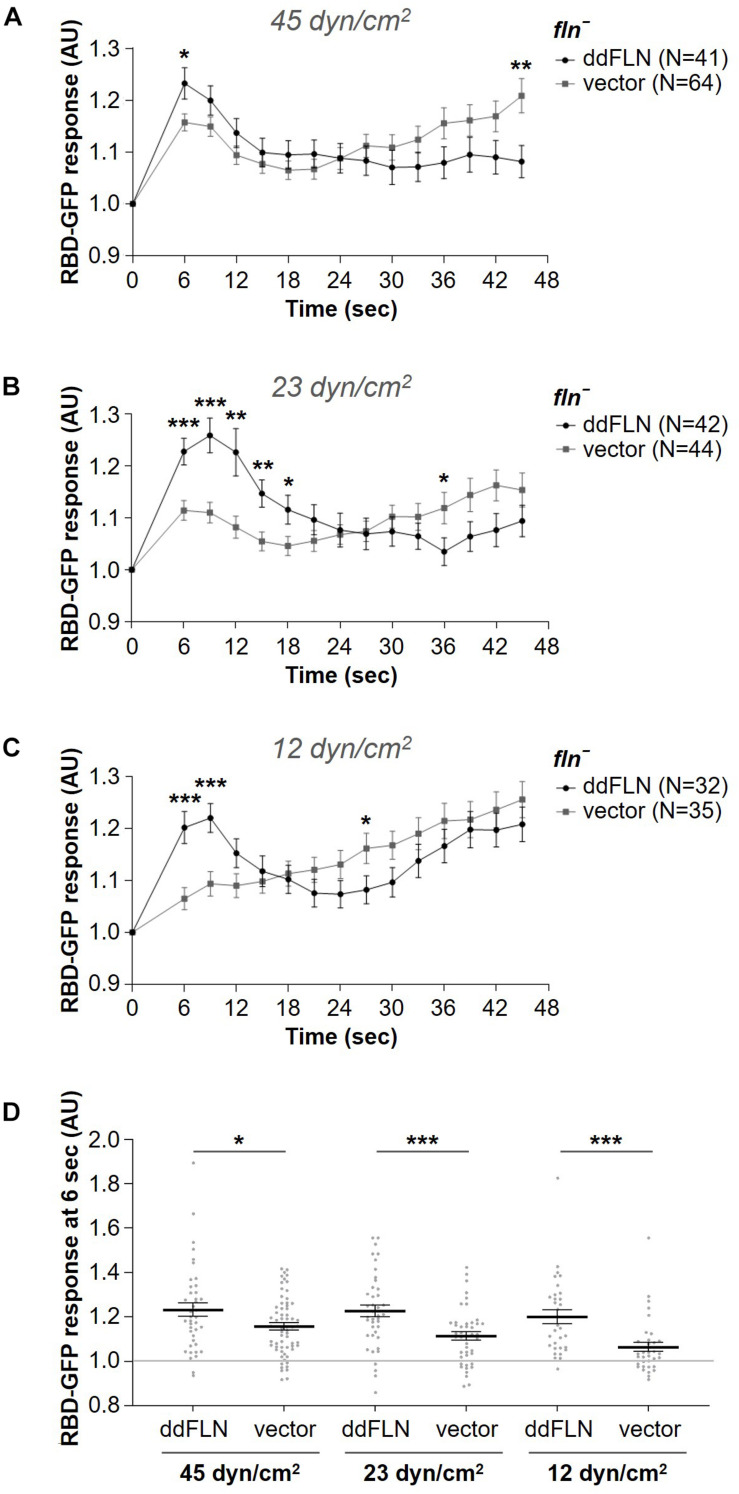
Filamin is required for optimal activation of the signal transduction network in response to acute stimulation with shear flow. **(A–C)** Activation of Ras, which reflects activation of the signal transduction network, was measured by the relocalization of RBD-GFP from the cytosol to the cell cortex. *fln*^–^ cells expressing RBD-GFP and either mCherry-tagged ddFLN or empty vector were imaged every 3 s by epifluorescence microscopy with a GFP filter set under 630X magnification with oil immersion. Shear flow was applied at time 0 for 2 s. The shear stress delivered to the cells was 45 dyn/cm^2^ **(A)**, 23 dyn/cm^2^ **(B)**, or 12 dyn/cm^2^ **(C)**. Data is displayed as mean ± SE. Number of cells analyzed is provided in the key and was pooled from four **(A)** or three **(B, C)** independent experiments. **(D)** RBD-GFP response values for individual cells from panels **(A–C)** at the 6 s time point. Horizontal lines with error bars correspond to mean ± SE. A line at the RBD-GFP response value of 1.0 is shown as a reference to indicate basal levels at time 0. *P < 0.05, **P < 0.01, and ***P < 0.001.

To test if filamin is required for the response to mechanical perturbation, we assessed spatiotemporal activation of Ras using the Ras-binding domain (RBD) biosensor, which has been previously shown to detect transient activation of Ras on the cortex following brief stimulation with shear flow (Artemenko et al., 2016). We used cells that lack filamin and express either empty vector (*fln*^–^) or mCherry-ddFLN (rescue), as well as GFP-tagged RBD. Following 2 s stimulation with shear flow at 45 dyn/cm^2^, rescue cells showed the expected peak of RBD accumulation at the cortex at around 6 s (Figure 2A). Although *fln*^–^ cells expressing an empty vector also showed a robust transient RBD response following stimulation, the peak response was significantly reduced compared to rescue cells by 32% (0.16 ± 0.02 vs. 0.23 ± 0.03 response over basal for *fln*^–^ vs. rescue cells, respectively; mean ± SE; *P* < 0.05; Figure 2D).

Since 45 dyn/cm^2^ is a strong stimulus that might mask the reduced sensitivity of *fln*^–^ cells, we also tested RBD-GFP response in *fln*^–^ and rescue cells following exposure to weaker shear forces, although the time of stimulation remained brief (2 s). At 23 dyn/cm^2^, the response of rescue cells was still very robust, although the peak shifted to 9 s (Figure 2B). The response of *fln*^–^ cells was even further reduced and was significantly different from the rescue both at 6 and 9 s, with a decrease of 50% for the 6 s peak (0.11 ± 0.02 vs. 0.23 ± 0.03 response over basal for *fln*^–^ vs. rescue cells, respectively; mean ± SE; *P* < 0.001; Figures 2B, D). Finally, at 12 dyn/cm^2^ the difference in the response was even more pronounced at 6 s with a 68% reduction in the response for *fln*^–^ vs. rescue cells (0.06 ± 0.02 vs. 0.20 ± 0.03 response over basal for *fln*^–^ vs. rescue cells, respectively; mean ± SE; *P* < 0.001; Figures 2C, D and Supplementary Video 2). A somewhat surprising observation was that while the response of *fln*^–^ cells was reduced at subsequently lower shear stress values, the response of rescue cells remained relatively constant, suggesting rescue cells might have increased sensitivity to mechanical stimulation. Overall, this suggests that ddFLN plays a role in the cell’s ability to respond to brief mechanical perturbation.

### Reduced Response of Cells Lacking ddFLN to Mechanical Stimulation Is Not Due to Overall Disruption of the Signal Transduction Network

**FIGURE 3:**
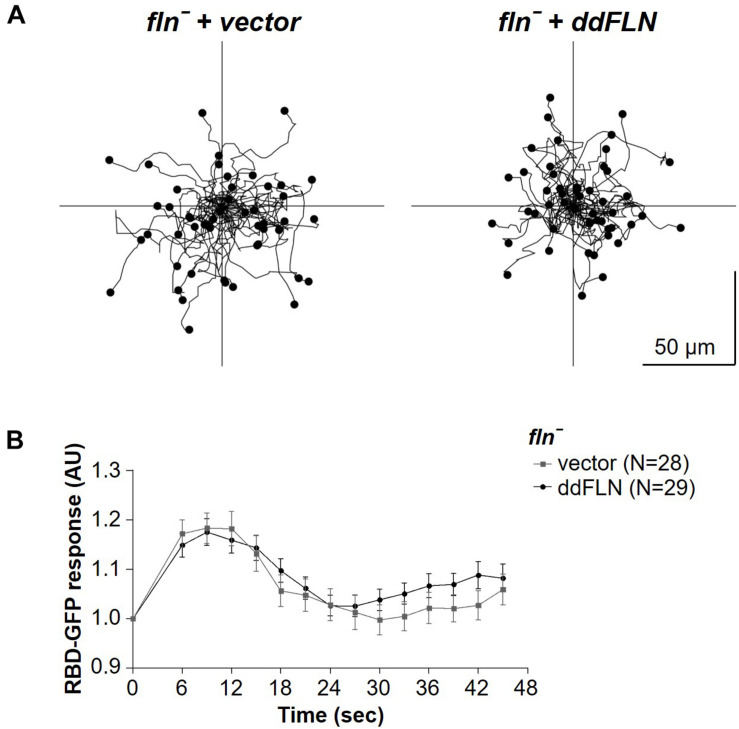
Response to chemical stimulation and random motility is not affected by lack of filamin. **(A)** *fln*^–^ cells expressing RBD-GFP and either mCherry-tagged ddFLN or empty vector were imaged using brightfield illumination under 200X magnification every 10 s as they were migrating without any stimulation. Tracks of 50 individual cells for each condition from one representative experiment are shown. Average data from four separate experiments is shown in Table 1. **(B)** The same cells as in panel **(A)** were stimulated with 100 μM folic acid at time 0. Response was measured as an inverse of drop in cytosolic intensity of RBD-GFP. Data is shown as mean ± SE. Number of cells analyzed is provided in the key and was pooled from four (ddFLN) or three (vector) separate stimulations.

We noticed that *fln*^–^ cells tended to have increased cortical RBD localization following the shut-off after the initial response to shear flow stimulation, suggesting that, perhaps, these cells generate more protrusions and/or become more motile compared to cells rescued with mCherry-ddFLN following stimulation. To check if *fln*^–^ and rescue cells might have differences in their basal activity, which would explain their differential responsiveness to stimulation, we examined random motility of both cell lines (Figure 3A, Table 1, and Supplementary Video 3). However, the two cell lines had comparable random migration speeds of 10.6 ± 1.6 and 8.2 ± 1.3 μm/min for *fln*^–^ and rescue cells, respectively (mean ± SE; *n* = 4; *P* > 0.05). It should be noted that there was a very small, but statistically significant, difference in the directness of the two cell lines (0.54 ± 0.03 vs. 0.50 ± 0.03 for *fln*^–^ vs. rescue cells, respectively; mean ± SE; *n* = 4; *P* < 0.01).

**TABLE 1:** Random migration of *fln*^–^ and wild-type cells expressing mCherry-tagged ddFLN or empty vector.

Strain	Velocity (μm/min)	Directness
*fln*^–^ + ddFLN	8.2 ± 1.3^a^	0.50 ± 0.03
*fln*^–^ + vector	10.6 ± 1.6	0.54 ± 0.03**
*WT* + ddFLN	16.6 ± 1.1	0.489 ± 0.007
*WT* + vector	17.5 ± 1.1	0.477 ± 0.003

^a^Values are mean ± SE of three experiments with 50 cells analyzed and averaged for each experiment. Images were acquired every 10 s for 7 min and cells were tracked for the last 5 min. **P < 0.01 compared to *fln*^–^ + ddFLN cells.

We also tested the response of these cells to a chemoattractant; however, global stimulation with folic acid resulted in the same magnitude of the RBD response in both rescue and *fln*^–^ cells (Figure 3B and Supplementary Video 4). This suggests that the reduced responsiveness of cells lacking ddFLN to mechanical cues is not due to a general decrease in the ability to activate the signal transduction network.

### Filamin Overexpression Slightly Improves the Response of the Signal Transduction Network to Mechanical Stimulation

**FIGURE 4:**
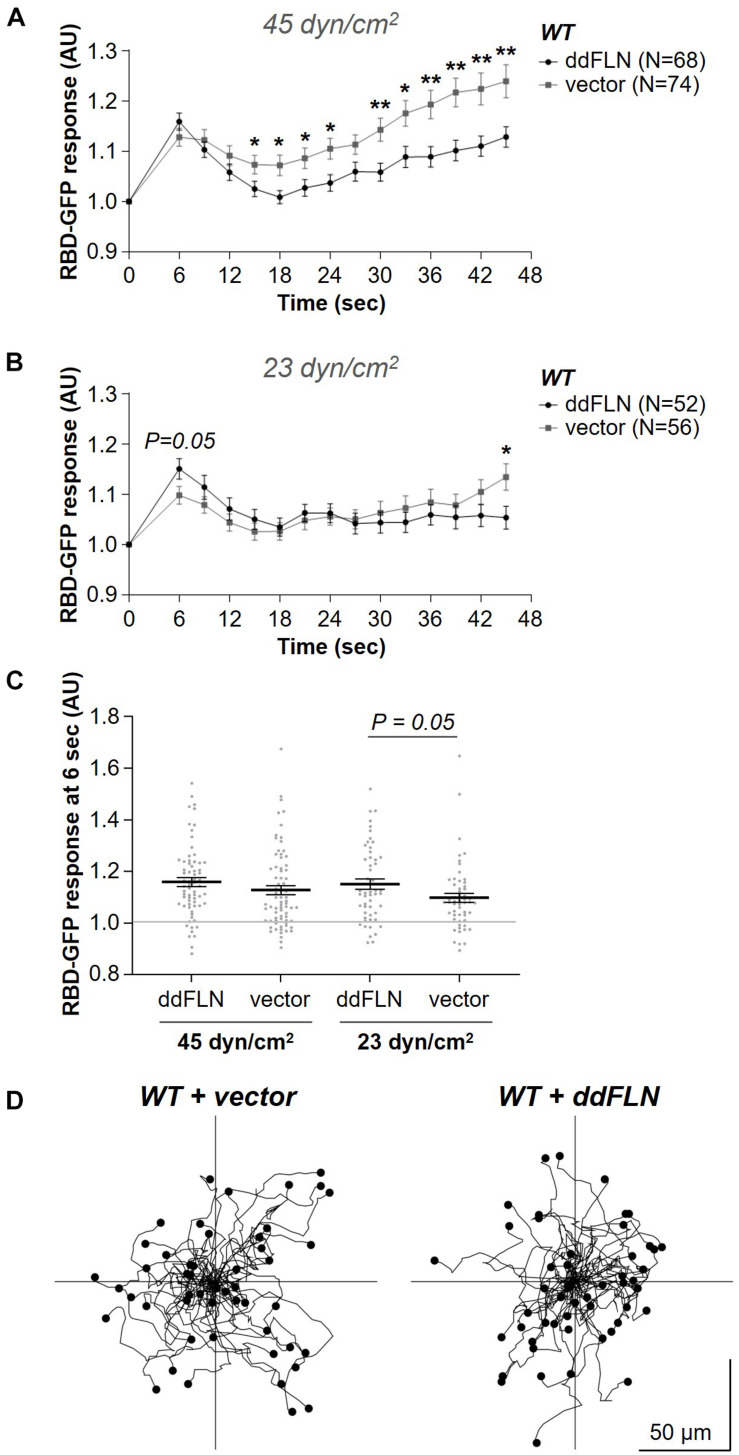
Overexpression of filamin slightly improves activation of the signal transduction network in response to shear flow without affecting the basal activity of the cells. **(A–C)** Activation of Ras, which reflects activation of the signal transduction network, was measured by the relocalization of RBD-GFP from the cytosol to the cell cortex. Vegetative wild-type (WT) cells expressing RBD-GFP and either mCherry-ddFLN or empty vector were imaged every 3 s by epifluorescence microscopy with a GFP filter set under 630X magnification with oil immersion. The shear stress delivered to the cells was 45 dyn/cm^2^ **(A)** or 23 dyn/cm^2^ **(B)**. Data is displayed as mean ± SE. Number of cells analyzed is provided in the key and was pooled from four **(A)** or three **(B)** independent experiments. **(C)** RBD-GFP response values for individual cells from panels **(A,B)** at the 6 s time point. Horizontal lines with error bars correspond to mean ± SE. A line at the RBD-GFP response value of 1.0 is shown as a reference to indicate basal levels at time 0. *P < 0.05, **P < 0.01. **(D)** The same cells as in panels **(A–C)** were imaged as they were migrating without any stimulation using brightfield illumination under 200X magnification every 10 s. Tracks of 50 individual cells for each condition from one representative experiment are shown. Average data from three separate experiments is shown in Table 1.

It is possible that expression of ddFLN in *fln*^–^ cells improved the response because of increased levels of ddFLN compared to endogenous levels in wild-type cells. To address this, we expressed mCherry-tagged ddFLN in wild-type cells and assessed their responsiveness to acute shear flow stimulation. Following application of the strong 45 dyn/cm^2^ shear stress stimulus, the initial response was comparable between wild-type cells expressing empty vector and mCherry-tagged ddFLN (Figures 4A,C). However, at the lower shear stress of 23 dyn/cm^2^ the initial response of overexpressors at 6 s post-stimulation was slightly stronger compared to cells with endogenous levels of ddFLN, although this difference did not quite reach statistical significance (0.10 ± 0.02 vs. 0.15 ± 0.02 response over basal for wild-type vs. overexpressor cells, respectively; mean ± SE; *P* = 0.05; Figures 4B,C). Interestingly, similarly to the observations in rescue cells in Figures 2A–C, overexpression of ddFLN in wild-type cells also appeared to reduce the secondary cortical RBD accumulation after the initial shut-off with significant differences found for most time points between 15 and 45 s following stimulation (Figure 4A). This was not likely due to differences in the overall basal activation state of the cells since random motility was comparable between the two cell lines (Figure 4D, Table 1, Supplementary Video 5).

### Filamin’s Actin-Binding Domain Is Required for Its Role in Shear Flow-Induced Response

**FIGURE 5:**
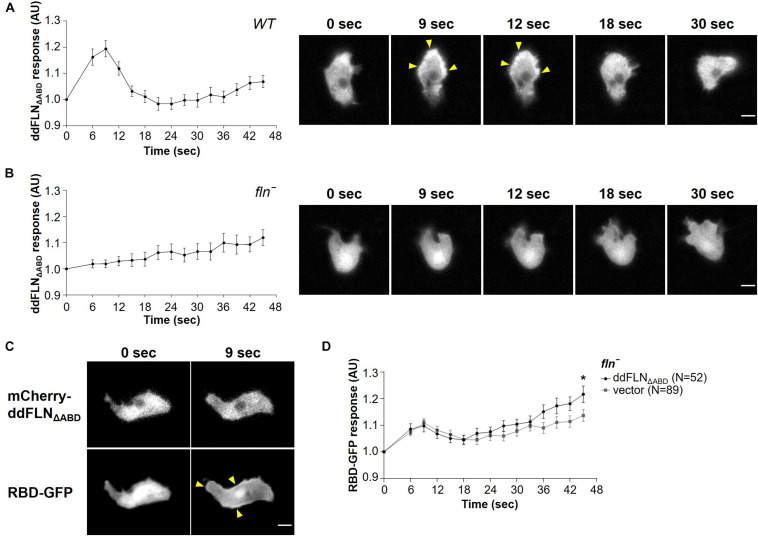
Filamin’s actin-binding domain is required for its role in shear flow-induced response. **(A–C)** Wild-type (WT) **(A)** or *fln*^–^ **(B)** cells expressing RBD-GFP and mCherry-tagged ddFLN_ΔABD_ were imaged every 3 s by epifluorescence microscopy with an RFP filter set under 630X magnification with oil immersion. Shear flow (45 dyn/cm^2^) was applied at time 0 for 2 s. Response was measured as protein relocalization from the cytosol to the cell cortex. Data is displayed as mean ± SE of 42 **(A)** or 30 **(B)** cells pooled from two independent experiments. A representative cell is shown to the right of the graph. **(C)** A representative *fln*^–^ cell expressing RBD-GFP and mCherry-tagged ddFLN_ΔABD_ is shown to demonstrate the lack of ddFLN _ΔABD_ translocation even in cells that have robust RBD-GFP recruitment to the cortex. Arrowheads point to biosensor accumulation at the cortex. Scale bar, 5 μm.**(D)** Activation of Ras, which reflects activation of the signal transduction network, was measured by the relocalization of RBD-GFP from the cytosol to the cell cortex in *fln*^–^ cells expressing RBD-GFP and either mCherry-tagged ddFLN_ΔABD_ or empty vector. Cells were imaged as above with a GFP filter set every 3 s and stimulated with 2 s of shear flow (45 dyn/cm^2^) at time 0. Data is displayed as mean ± SE. Number of cells analyzed is provided in the key and was pooled from three independent experiments. *P < 0.05.

To begin understanding how ddFLN is able to regulate cell response to shear flow, we generated mCherry-tagged ddFLN that lacks its actin-binding domain (ddFLN_ΔABD_). Surprisingly, ddFLN_ΔABD_ was still transiently recruited to the cortex of wild-type cells following 2 s stimulation with shear flow at 45 dyn/cm^2^ (Figure 5A and Supplementary Video 6). Since this relocalization could be mediated by dimerization of ddFLN_ΔABD_ with endogenous ddFLN, we also tested ddFLN_ΔABD_ response in *fln*^–^ cells. In the absence of endogenous ddFLN, there was no change in localization of ddFLN_ΔABD_ following shear flow stimulation (Figure 5B and Supplementary Video 6). Since overall signal transduction network response to shear flow is reduced in *fln*^–^ cells, we observed the behavior of ddFLN_ΔABD_ concomitantly with RBD-GFP; however, even when RBD-GFP translocation was clearly observed, ddFLN_ΔABD_ failed to relocalize in response to acute shear flow stimulation (Figure 5C). Thus, ddFLN accumulation at the cortex is likely mediated *via* its interaction with the actin cytoskeleton.

Next, we examined whether ddFLN_ΔABD_ can rescue the impaired response of *fln*^–^ cells to acute mechanical stimulation by examining localization of active Ras using the RBD-GFP biosensor (Figure 5D). As expected, *fln*^–^ cells expressing ddFLN_ΔABD_ had a similar response to cells expressing empty vector, confirming that ddFLN requires its ability to interact with the actin cytoskeleton for its role in sensing or transmitting mechanical stimuli.

### Lack of ddFLN Leads to Impaired Shear Flow-Induced Migration

**FIGURE 6:**
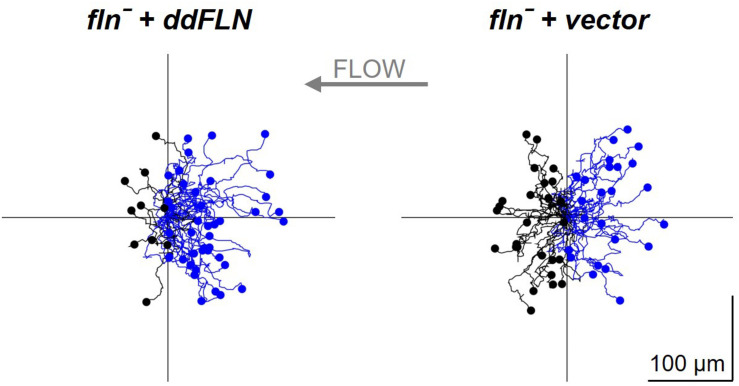
Filamin is required for shear flow-induced migration. *fln*^–^ cells expressing RBD-GFP and either mCherry-tagged ddFLN or empty vector were subjected to continuous flow at 6 dyn/cm^2^ and were imaged using brightfield illumination under 200X magnification every 10 s for 20 min. Individual cells were tracked starting 2 min after initiation of flow for the remaining 18 min. Tracks of 50 individual cells for each condition from one representative experiment are shown. Cells that showed displacement in the direction opposite of the flow are shown in blue; the rest are in black. Average data for cell speed and directionality from three separate experiments is shown in Table 2.

Since ddFLN appears to be important for the activation of the signal transduction network in response to shear flow, we next wanted to test if it is required for shear flow-induced migration. We first subjected wild-type cells to a variety of shear forces to determine the optimal conditions for examining shear flow-induced migration. As can be seen in Supplementary Video 7, wild-type cells migrated upstream at shear stress values of 6 and 12 dyn/cm^2^, but began losing direction and started detaching by 17 dyn/cm^2^. We chose to perform subsequent migration analysis at 6 dyn/cm^2^ since this condition resulted in robust migration against the flow without loss of cells by detachment. Similarly to wild-type cells, *fln*^–^ cells transformed with mCherry-tagged ddFLN showed a clear preference for migration against shear flow (Figure 6, Table 2, andSupplementary Video 8). On the other hand, *fln*^–^ cells appeared to be migrating more randomly, which was reflected in a significant reduction in the forward migration index compared to the rescue cells (0.1 ± 0.2 vs. 0.5 ± 0.1 for *fln*^–^ vs. rescue, respectively; *n* = 3; *P* < 0.05). Interestingly, the velocity of the two cell lines was comparable under continuous flow, although somewhat lower than when cells were migrating randomly (Table 1), suggesting the ddFLN plays a specific role in determining the direction of migration in response to a mechanical stimulus rather than affecting overall motility.

**TABLE 2:** Shear flow-induced directed migration of *fln*^–^ cells expressing mCherry-tagged ddFLN or empty vector.

Strain	Velocity (μm/min)	Directness	Forward migration index
*fln*^–^ + ddFLN	7.4 ± 0.4^*a*^	0.51 ± 0.02	0.5 ± 0.1
*fln*^–^ + vector	6.1 ± 1.2	0.48 ± 0.01	0.1 ± 0.2*

^*a*^Values are mean ± SE of three experiments with 50 cells analyzed and averaged for each experiment. Images were acquired every 10 s for 20 min while cells were continuously exposed to 6 dyn/cm^2^ shear stress. Cells were tracked for the last 18 min for analysis. *P < 0.05 compared to *fln*^–^ + ddFLN cells.

### α-Actinin Is Not Required for Optimal Response to Acute Mechanical Stimulation

**FIGURE 7:**
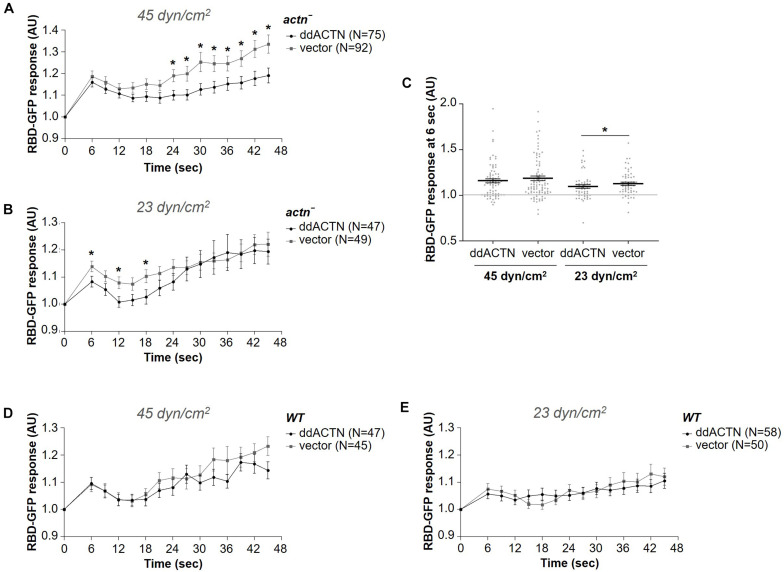
α-actinin is not required for the activation of the signal transduction network in response to shear flow. Activation of Ras, which reflects activation of the signal transduction network, was measured by the relocalization of RBD-GFP from the cytosol to the cell cortex. *actn*^–^ **(A–C)** or wild-type (WT) **(D,E)** cells expressing RBD-GFP and either mCherry-tagged ddACTN or empty vector were imaged every 3 s by epifluorescence microscopy with a GFP filter set under 630X magnification with oil immersion. Shear flow was applied at time 0 for 2 s. The shear stress delivered to the cells was 45 dyn/cm^2^ **(A,D)** or 23 dyn/cm2 **(B,E)**. Data is displayed as mean ± SE. Number of cells analyzed is provided in the key and was pooled from seven **(A)** or three **(B,D,E)** independent experiments. **(C)** RBD-GFP response values for individual cells from panels **(A,B)** at the 6 s time point. Horizontal lines with error bars correspond to mean ± SE. A line at the RBD-GFP response value of 1.0 is shown as a reference to indicate basal levels at time 0. *P < 0.05, **P < 0.01.

Since *fln*^–^ cells were still able to mount a response to acute mechanical stimulation, it is likely that other actin crosslinking proteins might be involved in sensing and/or transmitting the mechanical stimulus to downstream activation of the signal transduction network. Since α-actinin is a known mechanosensor in other systems (Luo et al., 2013; Schiffhauer et al., 2016; Le et al., 2017) and is recruited to the cortex following exposure to a brief shear flow stimulus (Figure 1B), we tested whether this actin-binding protein is necessary for the activation of the signal transduction network in response to shear flow similarly to ddFLN. Cells lacking ddACTN (*actn*^–^) with RBD-GFP and mCherry-tagged ddACTN or empty vector were subjected to a 2 s shear flow stimulation; however, both *actn*^–^ and rescue cells showed similar levels of Ras activation at 45 dyn/cm^2^ shear stress (Figures 7A, C). Notably, despite a similar initial response, *actn*^–^ cells appeared to have increased cortical RBD signal compared to rescue cells at later time points after the stimulation (*P* < 0.05 at all time points between 24 and 45 s), although the significance of this finding is unclear. Surprisingly, at the lower 23 dyn/cm^2^ shear stress level, *actn*^–^ cells had a slightly better response than rescue cells (0.13 ± 0.02 vs. 0.08 ± 0.02 response over basal at 6 s post-stimulation for *actn*^–^ vs. rescue cells, respectively; mean ± SE; *P* < 0.05; Figures 7B, C). This could mean that ddACTN is a negative regulator of the response. However, when we overexpressed ddACTN in wild-type cells, the RBD response was similar to cells with endogenous levels of ddACTN at both 45 and 23 dyn/cm^2^ (Figures 7D, E). Thus, ddACTN is likely not required for the cell’s ability to respond to acute mechanical stimulation.

## Discussion

Our study demonstrated that actin crosslinkers filamin and α-actinin play differential roles in the cell’s ability to respond to mechanical stimulation delivered by shear flow. Although both of these crosslinkers have direct mechanosensitive properties, only filamin appeared to be involved in the context of shear flow-induced activation of the signal transduction network and subsequent migration against the direction of flow. Not surprisingly, this function of filamin depended on its ability to bind to actin as evidenced by the lack of rescue of the reduced response in *fln*^–^ cells by ddFLN without ABD. The residual ability of cells without ddFLN to respond to shear flow suggests that other actin-binding proteins likely contribute to sensing and/or transmitting the response.

Both filamin and α-actinin were rapidly and transiently recruited to the cortex following mechanical stimulation with the same dynamics as recruitment of other leading edge markers, such as Ras or PI3K (Artemenko et al., 2016). This is consistent with the recruitment of these proteins to the pseudopods (Washington and Knecht, 2008) and is likely driven by their association with the newly polymerizing actin at the cortex. This is supported by the finding that ddFLN without ABD is not recruited except in wild-type cells, where it can presumably dimerize with the endogenous ddFLN that is recruited to the actin cortex. It would be interesting to determine whether ddFLN function in mechanotransduction observed here depends on the newly recruited ddFLN or on ddFLN that is basally present at the cortex. Given that the peak recruitment is observed at 6–9 s, it is likely that the initial sensing or transmission of the stimulus would occur prior to the bulk accumulation of ddFLN, which likely just follows actin polymerization, although it is possible that the stabilization of the actin network by newly recruited ddFLN is what is required to efficiently transmit the stimulus.

It is unclear whether filamin is directly involved in sensing or transmitting the mechanical stimulus in this system or if it simply affects the overall cortical tension, which in turn alters the ability of a cell to sense and/or respond to the mechanical stimulus. However, it seems unlikely that reduced cortical tension overall is responsible for the decreased initial response of cells lacking ddFLN since cortical tension is comparable or even slightly lower in cells lacking ddACTN (Luo et al., 2013), yet α-actinin-null cells had the same or even slightly improved response to shear flow stimulation. It should be noted that in the Luo et al. (2013) study, cortical tension was measured on cells in suspension, and it is possible that cells lacking ddFLN or ddACTN have differences in their cortical properties when they are adhered to a substrate. Both filamin and α-actinin participate in the formation of adhesion complexes and their linkage to the actin cytoskeleton in mammalian cells (Han and de Rooij, 2016). Although *D. discoideum* cells do not form focal adhesions similarly to other cells undergoing amoeboid-type migration, they nonetheless have specific adhesion molecules, such as SibA (Cornillon et al., 2006), that could potentially engage with ddFLN and/or ddACTN and contribute to cortical stiffness. This possibility needs to be further explored.

Although ddACTN did not appear to be required for the initial response of the signal transduction network to the shear flow stimulus, it may still play a role in the response since cells lacking ddACTN had improved Ras activation at the cortex. Although it is possible that ddACTN somehow negatively regulates the system, this is unlikely since addition of ddACTN did not adversely affect the initial response of wild-type cells. A more likely possibility is that cells without ddACTN might have a compensatory increase in ddFLN, or other crosslinkers, which could improve the response as was shown in this study for ddFLN. Other studies have suggested functional redundancy between ddFLN and ddACTN, since only knockout of both of these actin crosslinkers leads to severe defects in cell behavior and these defects can be rescued by the addition of one or the other protein (Witke et al., 1992; Rivero et al., 1996, 1999). However, since only *fln*^–^ cells had a clear defect in the response to shear flow stimulation in our study, it seems likely that ddFLN and ddACTN have non-redundant roles in mechanosensation and/or mechanotransduction that leads to shear flow-induced migration.

An interesting observation was that cells expressing ddFLN, either in the *fln*^–^ or wild-type background, appeared to remain less active, as judged by Ras activation at the cortex, for the duration of the assay following the initial response. This finding was somewhat surprising since we did not notice an overt reduction in cell activity by visual inspection of the videos, nor did we see a change in the basal activity of the cells based on random migration. However, it is possible that since ddFLN improved the initial response of the cells to the shear flow stimulus, this resulted in more efficient activation of the delayed negative feedback loop that turns off the network as is proposed by the STEN-CEN model (Pal et al., 2019). Alternatively, reduced Ras activity of ddFLN-expressing cells after the initial peak could be due to a general increase in cortical tension in these cells. This is supported by the observation that *actn*^–^ cells expressing ddACTN also showed significantly reduced RBD accumulation at the cortex between 24 and 45 s after stimulation, despite having no or inhibitory effects on the initial response. It would be interesting to examine whether cortical tension plays a role in a cell’s ability to resume its activity following global stimulation with a mechanical cue. Finally, accumulation of actin crosslinkers could also lead to increased bearing of the load from shear force, which would reduce further responsiveness, and might be especially relevant in cases of continuous stimulation with shear flow.

Cells expressing ddFLN responded strongly regardless of the stimulus strength, which ranged between 12 and 45 dyn/cm^2^, even though we previously showed that wild-type cells are able to distinguish between these shear stress levels and respond progressively better (Artemenko et al., 2016). Indeed, in this study, wild-type, ddFLN-null, and ddACTN-null cells showed a stronger response at higher shear stress levels. In contrast, cells expressing mCherry-ddFLN appeared to have a lower threshold for activation and responded equally strongly at all shear levels tested. Such improved sensitivity would give cells an advantage during continuous shear flow application that leads to directed migration. As the side of the cell facing the flow gets stimulated, the activation of the signal transduction network would bias actin polymerization in the direction of the gradient. Although this may suggest that the observed defect in the response of *fln*^–^ cells only appears as a defect because it is compared to the improved response of cells overexpressing ddFLN, this is unlikely since *fln*^–^ cells failed to sense direction during continuous shear flow application under conditions when wild-type cells efficiently migrate against the flow.

The upstream migration of cells in this study is consistent with our previous observations of vegetative wild-type cells grown in association with bacteria, which migrated against the flow at 10 dyn/cm^2^ but switched their direction at 25 dyn/cm^2^ (Artemenko et al., 2016). A study by Fache et al. (2005) also noted that *D. discoideum* cells migrate against the flow at lower shear stresses, although in that study cells switched to migration with the flow at shear stresses > 0.6 Pa (6 dyn/cm^2^). The discrepancy in the shear stress values that the cells can withstand and actively migrate toward is likely due to differences between cells grown axenically or in association with bacteria. Regardless of the specific shear stress values that induce directed migration in different cell types, it would be interesting to examine whether the underlying mechanisms that allow the cells to sense and orient themselves to face the flow are conserved and if filamin plays a similar role in shear flow-induced migration of *D. discoideum* at other stages of development or in other cell types.

It remains unclear whether filamin is a primary sensor of the mechanical stimulus or is an intermediate link between the sensor and signal transduction network activation. Physical force that transiently deforms the actin network could lead to a conformational change in filamin and expose cryptic binding sites for molecules that could transmit the stimulus to activate Ras or another component of the signal transduction or cytoskeletal machinery, causing firing of the entire network due to the presence of multiple feedback loops. Many candidate molecules have been identified as binding partners for filamin in mammalian cells, including R-Ras, Rac1 and RhoA (Lamsoul et al., 2020), although this remains to be examined in *D. discoideum* specifically in response to shear flow stimulation. The ability of filamin to accumulate in zones of high shear stress (Luo et al., 2013), makes filamin a good candidate for being the primary sensor in this system. Indeed, the differential involvement of filamin and α-actinin in mediating shear flow-induced cell migration is consistent with the differences in the mechanical properties of the two actin crosslinkers, and, more specifically, with how they directly respond to mechanical forces. Luo et al. (2013) demonstrated that while ddACTN accumulates in response to dilational stress generated by micropipette aspiration, ddFLN is observed at the neck of the micropipette, where it responds to shear deformation. Intriguingly, mammalian filamin B, but not filamin A, shows accumulation in response to shear deformation (Schiffhauer et al., 2016), suggesting that if filamin plays a conserved role in shear flow-induced migration in mammalian cells, it may do so in an isoform-specific manner.

An alternative scenario would be that another primary sensor, such as an adhesion molecule or a mechanosensitive ion channel, recruits filamin, which then transmits the signal downstream. Savinko et al. (2018) demonstrated that filamin is important for shear force-mediated increase in adhesion *via* integrins during shear flow-induced migration of T lymphocytes. However, it remains unclear whether filamin plays a similar role in *D. discoideum* cells, which do not rely on integrin-dependent adhesion (Sebe-Pedros et al., 2010; Loomis et al., 2012; Kamprad et al., 2018). Additionally, a cation channel PKD2 has been implicated in shear flow motility in *Dictyostelium* (Lima et al., 2014) and filamin has been shown to associate with PKD2 homolog polycystin-2 in mammalian cells (Wang et al., 2015). Although PKD2 did not appear to be involved in the initial response of cells to acute stimulation with shear flow (Artemenko et al., 2016), it is possible that under more stringent conditions, such as reduced shear stress levels, cells lacking PKD2 would show defects in their ability to respond to mechanical cues. Future studies should focus on determining whether ddFLN associates with unique partners following stimulation with shear flow to figure out how it is able to alter cell response to this type of stimulus.

## Data Availability Statement

The raw data supporting the conclusions of this article will be made available by the authors, without undue reservation.

## Author Contributions

AC contributed to experimental design, performed experiments and data analysis, and wrote sections of the manuscript. SB performed experiments and data analysis. JM acquired and analyzed preliminary data and contributed to experimental design. YA conceived the study and experimental design, supervised the project, and wrote the manuscript. All authors read and approved the submitted manuscript.

## Conflict of Interest

The authors declare that the research was conducted in the absence of any commercial or financial relationships that could be construed as a potential conflict of interest.

## Publisher’s Note

All claims expressed in this article are solely those of the authors and do not necessarily represent those of their affiliated organizations, or those of the publisher, the editors and the reviewers. Any product that may be evaluated in this article, or claim that may be made by its manufacturer, is not guaranteed or endorsed by the publisher.
