# Supplementary material for: Differential Roles of Actin Crosslinking Proteins Filamin and α-Actinin in Shear Flow-Induced Migration of Dictyostelium discoideum
Source: Front Cell Dev Biol. 2021 Aug 16;9:743011. doi: 10.3389/fcell.2021.743011 (PMC8415421; doi:10.3389/fcell.2021.743011)
Supplement: Supplementary file 9 [file Data_Sheet_1.PDF]

Supplementary Material

Supplemental Video Legends

Supplemental Video S1. ddFLN and ddACTN localization in response to acute treatment with shear flow. Wild-type cells expressing mCherry-tagged ddACTN or mCherry-tagged ddFLN were imaged every 3 sec for 20 frames by epifluorescence microscopy with an RFP filter set under 630X magnification with oil immersion. Shear flow at 45 dyn/cm^2 pressure was applied at time 0 for 2 sec. Playback speed is two frames per second. Movie corresponds to Figures 1A and 1B.

Supplemental Video S2. RBD-GFP response to acute stimulation with shear flow in cells with and without ddFLN. *fln*$^-$ cells expressing RBD-GFP and either mCherry-ddFLN or vector were imaged every 3 sec for 20 frames by epifluorescence microscopy with a GFP filter set under 630X magnification with oil immersion. Shear flow at 12 dyn/cm^2 pressure was applied at time 0 for 2 sec. The ddFLN cell shown here was positive for the mCherry signal. Playback speed is two frames per second. Movie corresponds to Figure 2C.

Supplemental Video S3. Random migration of cells with and without ddFLN. *fln*$^-$ cells expressing RBD-GFP and either mCherry-ddFLN or vector were imaged using brightfield illumination under 200X magnification every 10 sec for 5 minutes as they were migrating without any stimulation. Playback speed is ten frames per second. Movie corresponds to Figure 3A.

Supplemental Video S4. Folic acid response in cells with and without ddFLN. *fln*$^-$ cells expressing RBD-GFP and either mCherry-ddFLN or vector were imaged every 3 sec for 20 frames by epifluorescence microscopy with a GFP filter set under 630X magnification with oil immersion. 100 μM of folic acid was added at time 0. Both ddFLN cells shown here were positive for the mCherry signal. Playback speed is two frames per second. Movie corresponds to Figure 3B.

Supplemental Video S5. Random migration of wild-type cells with or without ddFLN overexpression. Wild-type cells expressing RBD-GFP and mCherry-ddFLN or vector were imaged using brightfield illumination under 200X magnification every 10 sec for 5 minutes as they were migrating without any stimulation. Playback speed is ten frames per second. Movie corresponds to Figure 4D.

Supplemental Video S6. ddFLN$_{\Delta ABD}$ localization in response to acute treatment with shear flow in wild-type and *fln*$^-$ cells. Wild-type or *fln*$^-$ cells expressing mCherry-ddFLN$_{\Delta ABD}$ were imaged every 3 sec for 20 frames by epifluorescence microscopy with an RFP filter set under 630X magnification with oil immersion. Shear flow at 45 dyn/cm^2 pressure was applied at time 0 for 2 sec. Playback speed is two frames per second. Movie corresponds to Figures 5A and 5B.

Supplemental Video S7. Directed migration of wild-type cells under continuous shear stress of 6, 12, or 17 dyn/cm^2. Wild-type cells were imaged using brightfield illumination under 200X magnification every 10 sec for 12 minutes as they were migrating under continuous flow delivered from right to left. Movie begins 2 minutes after the start of continuous stimulation. Playback speed is ten frames per second.

Supplemental Video S8. Directed migration of cells with and without ddFLN under continuous shear stress of 6 dyn/cm^2. *fln*$^-$ cells expressing RBD-GFP and either mCherry-ddFLN or vector were imaged using brightfield illumination under 200X magnification every 10 sec for 20 minutes as they were migrating with stimulation from right to left. Movie begins 2 minutes after the start of continuous stimulation. Playback speed is ten frames per second. Movie corresponds to Figure 6.
